# Evaluation of transdermal drug delivery using terahertz pulsed imaging

**DOI:** 10.1364/BOE.394436

**Published:** 2020-07-20

**Authors:** Jiarui Wang, Hannah Lindley-Hatcher, Kai Liu, Emma Pickwell-MacPherson

**Affiliations:** 1 Department of Electronic Engineering, The Chinese University of Hong Kong, Shatin, Hong Kong; 2 Department of Physics, University of Warwick, Coventry, UK

## Abstract

Transdermal drug delivery (TDD) is widely used for painless dosing due to its minimally invasive nature compared to hypodermic needle injection and its avoidance of the gastrointestinal tract. However, the stratum corneum obstructs the permeation of drugs into skin. Microneedle and nanoneedle patches are ways to enhance this permeation. In this work, terahertz (THz) imaging is utilized to compare the efficacy of different TDD methods including topical application and via a needle patch. Our work shows the feasibility and potential of using THz imaging to quantify and evaluate different transdermal application methods.

## 1. Introduction

Terahertz (THz) radiation lies between 0.1 to 10 THz and has a wide range of potential applications in biomedical areas [1]. Based on its non-invasive and non-ionizing nature, THz imaging is desirable for *in vivo* human diagnosis and monitoring. Research has shown that THz imaging is able to evaluate the water content of skin *in vivo* [2,3]. Hernandez-Cardoso *et al.* demonstrated that due to water content changes, THz imaging can be used to detect early signs of vascular deterioration caused by diabetic foot syndrome [4]. Wang *et al.* used THz imaging to monitor the effect of silicone gel on human skin and proved that THz radiation can be used for real-time monitoring of treatment efficiency [2]. Other work including burn scar imaging and corneal hydration sensing have also attracted interest for investigation with THz radiation [5,6]. Aside from *in vivo* measurements, *ex vivo* measurements of biomedical samples with THz imaging also play an important role in understanding the biological and medical mechanisms at work. He *et al*. measured dehydrated porcine samples extracted from different regions and applied effective medium theory to quantify the water concentration in fresh porcine skin [7], this is of particular interest due to the similarities between human and porcine skin. Woodward *et al*. used THz imaging to differentiate between basal cell carcinoma and healthy skin, this has the potential to aid surgery in the future [8]. Further research into the potential for *ex vivo* studies and endoscopic developments of THz systems will continue to broaden the range of applications in biomedical areas.

Transdermal drug delivery (TDD) has recently attracted interest as it is possible to avoid the gastrointestinal tract and is painless, unlike alternative drug application methods such as oral and hypodermic needle applications [9]. However, due to the resistance of the stratum corneum (SC), poor drug transport is limiting the development of TDD. Micro/Nano needle patches are one of the methods used to enhance TDD efficiency by creating small conduits in the SC to enable transport through the bio-membrane. Microneedle patches are arrays of needles on the micro scale which can easily penetrate the SC and deliver drugs to the epidermis or dermis layers in the skin without disturbing nerves. Solid microneedle patches are used to create temporary channels in the skin surface and after removing it, the drug is then applied directly to the target area allowing it to diffuse to inner layers of the skin through the created channels [10,11]. However, repeated applications of a microneedle patch may lead to skin irritation. Nanoneedles provide another option as they are extremely small [12]. To understand the drug delivery efficacy, different imaging methods have been used to reveal the TDD process. Apart from biopsy and histological staining, optical coherence tomography (OCT) and fluorescence imaging are two common methods to evaluate the efficiency of TDD [13,14]. OCT can be used to study the structure of the skin, and fluorescence imaging is able to analyze the penetration depth of fluorescent-labeled drugs. There are also other emerging techniques like photoacoustic microscopy and laser speckle contrast imaging in this area [13,15]. To further facilitate the study, label-free techniques that can provide *in vivo* imaging and can quantify drugs are in high demand.

THz imaging could be a way to help monitor the delivery of drugs *in vivo*. Its high sensitivity to liquids means it can easily track drugs dissolved in water, glycerol or other solutions. Moreover, it enables us to acquire the THz spectrum of the target area which would also assist in detecting characteristic peaks or troughs in the frequency domain for certain drugs. Kim *et al*. used THz imaging in an *ex vivo* study to monitor ketoprofen dissolved in dimethyl sulfoxide (DMSO) applied to the surface of an excised skin sample. Imaging in reflection geometry of the underside of the skin sample showed that the amount of drug that penetrates through skin can be determined by the THz signal [16]. Their further studies also show that THz imaging can even distinguish different concentrations of drugs on the skin surface [17]. To further utilize the spectrum information, Naccache et al. used the spectrum peak of ibuprofen at around 1.04 THz for THz thermometry and imaging [18].

Glycerol is routinely used as a component in cosmetic products [19] and Lashmar et al. show that 50% w/v glycerol/water mixture causes no discernible histological changes in nude mice skin [20]. Oh *et al.* used glycerol to enhance the THz imaging contrast [19]. Therefore, using an adequate concentration of glycerol/water mixture as the solvent can give a good contrast and in the meantime cause very little damage to skin. Aspirin is known as an antipyretic, analgesic and antiplatelet drug. However, oral administration of aspirin has gastrointestinal side effects [21]. Transdermal delivery provides a more convenient, safer alternative to avoid gastrointestinal side effects [21]. Here, in this work, we use THz imaging to detect transdermal aspirin solution delivery and compare different administration methods.

## 2. Methodology

### 2.1 THz system and experimental procedure

**Fig. 1.**
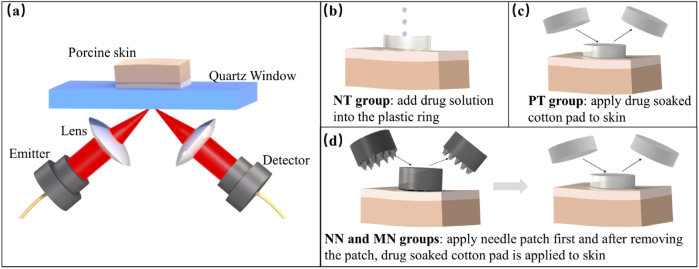
(a) Experimental setup for the imaging. Diagrams of the treatment process for the (b) NT group (c) PT group (d) NN and MN groups.

The Menlo TERA K15 THz time-domain system was chosen for this project. Figure 1(a) shows our reflection geometry setup which has a z-cut crystalline quartz window to help align the sample (porcine skin). THz light is focused onto the quartz-sample interface by the focal lens. The detector records the time-domain pulses and by using a Fourier Transform, the frequency domain information can also be obtained.

For our study, we synthesized a drug solution by adding 10% aspirin/ethanol (50 mg/ml) to a 50% w/v glycerol/water solution. The solution is used for transdermal drug delivery by different methods. Due to the potential risks of conducting *in vivo* measurements on humans, we have conducted *ex vivo* experiments on fresh porcine skin. Only hairless areas on the skin were chosen. The porcine skin was cut into 3×3 cm^2^ pieces and cleaned with a moist cotton pad. Cling film was then used to prevent the porcine skin from dehydrating before the THz measurements. In total, we divided the samples into 5 groups and for each group, we repeated the protocol 10 times. Table 1 and Fig. 1(b)-(d) detail the groups and their corresponding treatment protocols. For the treatment groups apart from NT, the drug-soaked cotton pads were applied to the porcine skin for 5 minutes, and then removed. This controlled the dose level so that the drug would be absorbed without leaving a residue. This was checked using a microscope. However for the NT group, the residue was wiped off prior to imaging. Each piece of porcine skin was imaged before and after applying any treatment. Each image is 2×2 cm^2^ with 0.5 mm^2^ for each pixel. After performing the measurement, data processing methods described below were used to analyze the results.

**Table 1.** Groups and Corresponding Treatment Protocols

Control	Normal Transdermal Drug Delivery (NT)	Transdermal Drug Delivery by Pad (PT)	Nano-Needle Drug Delivery (NN)	Micro-Needle Drug Delivery (MN)
No drug applied.	5 µl of the drug solution was applied to the surface of the porcine skin. A rubber ring was used to confine the liquid.	80 µl of the drug was dripped onto a 1 cm diameter cotton pad and then the pad was applied to the porcine skin surface.	A nano-needle patch (Nano needle, Konmison) was used to create nano-scale holes on the surface of the porcine skin and then a cotton pad with 80 µl drug was applied.	Same as NN but used a micro-needle patch (9-pin microneedle, Konmison) instead.

### 2.2 Data processing

There are two reflections in our measurement due to the use of quartz window: one from the air-quartz interface and one from the quartz-sample interface. Detailed information about eliminating the uneven thickness of the quartz window is previously reported in [22]. Air is measured as the reference. The sample to reference ratio (M) is defined as

(1) $$M\text{ = }\frac{FFT(E_{sample}(t)-E_{baseline}(t))}{FFT(E_{air}(t)-E_{baseline}(t))}\text{ = }\frac{r_{qs}}{r_{qa}}=\frac{{\tilde{n}}_{q}\cos\theta_{q}-{\tilde{n}}_{s}\cos\theta_{s}}{{\tilde{n}}_{q}\cos\theta_{q}+{\tilde{n}}_{s}\cos\theta_{s}}\cdot \frac{{\tilde{n}}_{q}\cos\theta_{q}+{\tilde{n}}_{a}\cos\theta_{a}}{{\tilde{n}}_{q}\cos\theta_{q}-{\tilde{n}}_{a}\cos\theta_{a}}$$

where $E_{sample}$ and $E_{air}$ are the reflection from sample and reference (air). Note that the reflection from the air-quartz interface is called the baseline ($E_{baseline}$) and is subtracted from the reflected signal of the sample ($E_{sample}$) and air ($E_{air}$). Then by applying the Fresnel equations in Eq. (1) and Snell’s Law given by

(2) $${\tilde{n}}_{a}\sin\theta_{a}={\tilde{n}}_{q}\sin\theta_{q}={\tilde{n}}_{s}\sin\theta_{s}$$

the refractive index of the sample ($\tilde{n_{s}}$) can be extracted from the measured sample to reference ratio (*M*). Note that ${\tilde{n}}_{q}$, ${\tilde{n}}_{a}$, $\theta_{q}$, $\theta_{s}$, $\theta_{a}$ are the refractive index of quartz and air and the incident angles in quartz, sample and air respectively. The refractive index is a complex number and its imaginary part is called the extinction ratio (*k*). The absorption coefficient (*α*) can be calculated via

(3) α = 4πfk/c

where *f* and *c* are the frequency and speed of light respectively.

## 3. Results and discussion

### 3.1 Drug spectrum

**Fig. 2.**
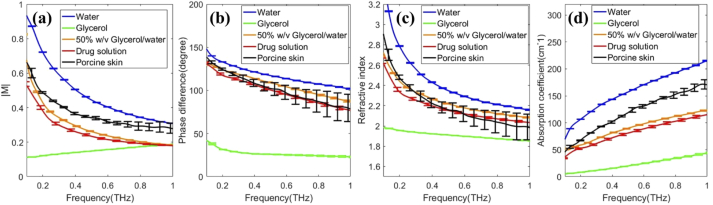
Spectra of different solutions and fresh porcine skin. (a) Amplitude and (b) Phase difference of M (c) Refractive index (d) Absorption coefficient of different solutions.

In Fig. 2, the frequency spectra of the drug solution and porcine skin are compared with water, glycerol and glycerol/water solution. Note that *M* in Eq. (1) is a complex number: in the following we show both the amplitude and phase information of *M*. In Fig. 2(a), from the amplitude spectrum data, we can see that the spectra of glycerol/water and drug solutions are significantly lower than porcine skin in the low frequency region due to the properties of glycerol solution. This result confirms the feasibility of using THz imaging to monitor drug solutions inside the skin. However, for the phase, the differences are small. For the refractive index, the drug solution shows little difference from the porcine skin but for the absorption coefficient, the difference is significant. However, at high frequencies, the standard deviation (error bar) of the porcine skin increases due to a possible air gap between the imaging window and the sample. The amplitude ratio (|M|) and the absorption coefficient (α) are significantly different for several frequencies and provide useful classification parameters.

### 3.2 Imaging results

Imaging results can give a better indication of how effective THz imaging is when applied to investigate TDD. However, to eliminate the effect of the variance between different pieces of porcine skin, the results after treatment need to be normalized to the original result before any treatment. Here, we first average the results before treatment from the central 25 points to get $\bar{{|M|}_{before}}$, this is used to normalize the result after the treatment ($|M{|}_{after}$) to get the normalized result as shown in Eq. (4).

(4) $$|M_{ij}{|}_{norm}=\frac{{|M_{ij}|}_{after}-\bar{{|M|}_{before}}}{\bar{{|M|}_{before}}}$$

**Fig. 3.**
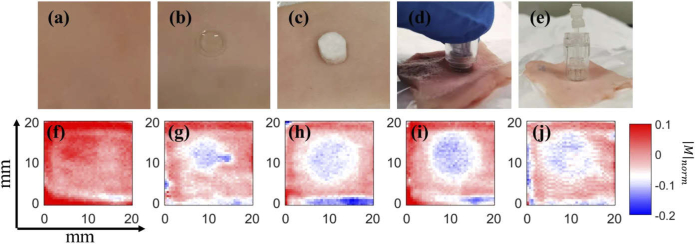
Imaging result of 5 groups (a,f) control group (b,g) NT group (c,h) PT group (d,i) NN group (e,j) MN group. $|M{|}_{norm}$ at 0.3 THz is plotted here. Table 1 specifies the treatment for each group.

Figure 3(a)-(e) show the different treatments carried out on fresh porcine skin and Fig. 3(f)-(j) show representative imaging results corresponding to the treatments. Figure 3(g)-(h) show that the $|M{|}_{norm}$ decreases in the site where the drug was applied. Though we cannot accurately map the amount of drug solution inside the skin from the current data, we can estimate the relative amount of drug delivered based on |M|. We expect that the drug solution will mainly displace water inside the skin and that the more solution delivered into the skin, the more $|M{|}_{norm}$ decreases. (This can be shown quantitatively using effective medium theory by setting the initial skin composition to be 30% biological background and 70% water, and replacing different proportions of water with drug solution.) However, if we first apply a nanoneedle patch onto the skin and then use the soaked pad for TDD, as shown in Fig. 3(i), $|M{|}_{norm}$ decreases more significantly compared to Fig. 3(g)–(h). This is due to increased permeability of the skin caused by the nanoneedle patch. The microneedle patch (Fig. 3(j)) does not show much improvement due to the small number of microneedles in one patch.

### 3.3 Data analysis

**Fig. 4.**
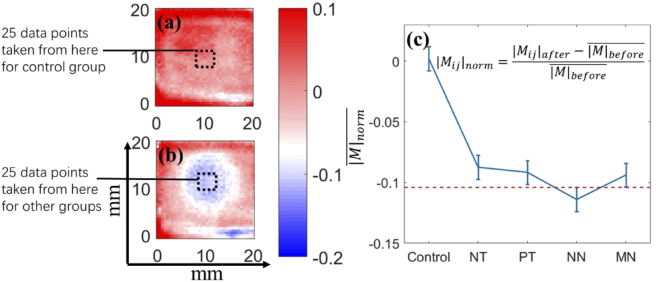
$|M{|}_{norm}$ at 0.3 THz from (a) the control group and (b) the NN treatment group. (c)Tukey’s Honestly Significant Difference Test of $\bar{{|M|}_{norm}}$. Error bars are Tukey’s minimal significant difference with α_Tukey_=0.05.

The 5 groups were repeated 10 times and the averages of $|M{|}_{norm}$ (represented by $\bar{{|M|}_{norm}}$) in the central 25 data points in the image/drug applied site for each group are used in the subsequent analysis. Figure 4(a)-(b) shows the representative $|M{|}_{norm}$ images of control and NN groups. The normality of $\bar{{|M|}_{norm}}$ is checked by the Shapiro-Wilk Normality test [23]. We use the one-way analysis of variance (ANOVA) and Tukey-Kramer test [24] in matlab to check for a significant difference between the groups. The results show that there is a significant difference between the groups (F=85.05, p<0.05). Figure 4(c) shows that after treatment all 4 groups are significantly different compared to the control group. However, $\bar{{|M|}_{norm}}$ of the NN group is significantly smaller compared to the other treatment groups which indicates that the nanoneedle patch can better increase the TDD efficiency compared to the other applications. Note that the error bars are not the standard deviation in each group but the Tukey’s minimal significant difference, calculated with the parameter α_Tukey_=0.05. The red dashed line indicates that the NN group does not overlap with any of the other groups. $\bar{{|M|}_{norm}}$ of the MN group is not as low as for the NN group due to the small number of microneedles in one patch.

## 4. Summary

In this work, we use THz imaging to monitor TDD via topical and micro/nano needle patches and compare the differences between application strategies. We also use several statistical methods to evaluate whether there is a significant difference between groups. Our work is the first attempt to compare different methods of TDD using THz radiation as a quantitative imaging technique. The results show that with a nanoneedle patch, the TDD is significantly increased compared to the other TDD approaches studied. However, further investigations are still needed for example to test the effect of different sizes of the molecules being applied and to accurately model the amount of drug permeated into skin. Ultimately we envisage that THz imaging could be used to monitor and evaluate TDD *in vivo*, and be useful for the development of TDD techniques and treatments.

## Data Availability

The datasets used in this work are available from https://figshare.com/articles/data_rar/12073566.
